# The Cholera

**Published:** 1848

**Authors:** 


### ARTICLE XI.

### *The Cholera.*

By the latest arrivals from Europe, which bring dates from London to the 20th of October, it appears that the cholera has not advanced in Great Britain with the rapid strides which have characterized its appearance in many cities on the Continent. We have condensed the returns from various places, and here give them.

In London, there were four cases of cholera reported on Monday (16th), and eleven on Tuesday, making fifteen cases in addition to those reported by the Register-General up to Saturday.

In Sunderland, another case of cholera was reported on Tuesday to the Customs by the medical staff appointed to inquire into the character and deaths on board of vessels in this port. In Edinburgh, Leith, and Newhaven, it is calculated that, since the cholera first broke out, the total number of cases that have appeared will amount to upwards of a hundred, and the number of deaths to about seventy. In Birmingham, a case of decided Asiatic cholera is said to have occurred on the 15th. Since Thursday, there have occurred in Hull nine cases of cholera, seven of which have proved fatal. Of these seven, two have occurred on board of vessels lying at the port, the remaining five in the town. Up to Thursday last there had been no death from Asiatic cholera in the town, the disease having, until that day, been confined to the vessels visiting the port.

*Prussia.*—A letter from Berlin, of the 13th instant, says—"The cholera report at this place, although showing a steady drain on the population, averaging from twenty-five to thirty cases daily, is less alarming than it was; but at Konigsberg the disease is making great ravages, and the cases, all of a most malignant character, amount to ninety or a hundred per day. Here the malady continues to attach itself to the humid and ill-ventilated portions of the town.

*Amsterdam.*—Letters from this city, dated Oct. 13th, state that several cases of Asiatic cholera have been declared in that city, some of which have terminated fatally. At Konigsberg (Prussia) the disease is raging fearfully, and up to the 10th instant 720 persons had been attacked, of whom 286 succumbed, and only 112 were cured.

*Quarantine.*—The British Government has resolved, on the recommendation of the Board of Health, to do away with the idle precaution of six days' quarantine, inflicted on continental vessels suspected of cholera.

Fortunately, the cholera has taken upon itself to give us proofs of its non-contagiousness; but admitting the doctrine of contagion to the utmost, how foolish and unfair would it be to persist in sending to a quarantine ground vessels from foreign parts that are merely suspected, whilst ships from our own ports, where cholera has manifested itself, are not interfered witn. The Royal Board of Trade at Stockholm, under date the 6th ult., has declared the port of Hull infected with the cholera, and all other ports in England and Wales suspected.

The quarantine of 7 and 14 days lately imposed at Naples, on vessels from Palermo and Malta, and which was suspected to have had only a political object, has been removed in conseuuence of the remonstrances of the British Admiral.

---

An order has been introduced into the Common Council of Boston, requesting the Consulting Physicians to advise the citizens in regard to proper measures, should the Asiatic cholera appear in the city.

The Councils of Washington City adopted resolutions, on Friday evening of last week (30th ult.), requesting the Board of Health to give general instructions on the subject, and to see that everything proper was done; also appointing a special committee to act in concert with the Board of Health.

By those who have been interested in the subject, it will be remembered that from England the cholera passed to Paris, and soon after entered this country b, the way of Canada; and all this within a few months.—*Bos. Med. and Surg. Jour.*

